# Teaching and Practicing Humanism and Empathy through Embodied Engagement

**DOI:** 10.3390/medicina58030330

**Published:** 2022-02-22

**Authors:** Sana Loue

**Affiliations:** Department of Bioethics, Case Western Reserve University School of Medicine, Cleveland, OH 44106, USA; sana.loue@case.edu

**Keywords:** embodiment, empathy, humanism, medical education

## Abstract

Concerns have been raised regarding medicine’s dehumanization of patients and providers and regarding the need to include, in the medical school curriculum, components that encourage the development of empathy and humanistic practice. This essay suggests that the development of humanistic practice requires attention to not only the cognitive and affective/emotive aspects of humanism, but also to the nurturing of intersubjectivity between the provider and the patient through strategies designed to promote embodied awareness. Several approaches to the development of embodied awareness are discussed, including puppetry pedagogy, drama, and virtual reality applications.

## 1. Introduction

The extant literature reflects a growing concern with respect to medicine’s dehumanization of patients and providers, such that patients are viewed as objectified bodies [1] or body parts [2] biomedicine reduces patients to bodies and body parts [3,4] clinical practice focuses on the organ specifics of disease [5,6,7] and the nonmaterial components of personhood remain unacknowledged and ignored [7,8]. Additional discussion has focused on the perception of professional competence as comprising solely or primarily the effective application of abstract knowledge [9,10] and technical expertise [11,12]. Emotional competence in the practice of medicine has been depicted as one of emotional distance between the patient and the provider [12], reflecting the longstanding view that it should be one of emotional “toughness” [1], affective neutrality [13], and detached concern [14,15].

More recently, efforts have been made to identify strategies that can be utilized to guide medical students toward a more humanistic, empathetic practice [16,17,18]. A large body of literature relating to medical education suggests that medical professionalism, comprising humanism’s cognitive components of knowledge, attitudes, and ethics [19] may serve as a pathway to the development of a humanistic practice. Montgomery, Loue, and Stange [20] concluded from their review of medical and health professionals education literature that the development of a humanistic practice also requires a focus on the emotive aspects of humanism—altruism, empathy, compassion, and caring [21,22,23,24]—and a connection and bidirectional flow between the cognitive (head) and emotive (heart) elements. Their nonlinear model drew from Wilbur’s four ways of knowing [25,26]: the interior of an individual; the exterior, referring to knowledge derived from observing individuals’ behavior in isolation; the collective interior, i.e., shared norms and values; and the collective exterior, or the observed behavior of a group.

Montgomery and colleagues [20] posited that humanism could be fostered through the creation of a flow between the cognitive and subjective domains within an individual, set into motion through the activation of any one of four levers: personal reflection, action, collective reflection, and system support. This essay builds upon the work of Montgomery and colleagues and others to suggest that the development of humanistic practice also requires the nurturing of intersubjectivity between the provider and the patient through strategies designed to promote embodied awareness. Accordingly, the discussion focuses, first, on defining embodiment and, second, on presenting educational interventions specifically designed to nurture intersubjectivity through embodied awareness.

## 2. Defining Embodiment

Despite the widespread use of the term “embodiment,” there is little consensus about its meaning [27]. In the context of context of medical care, the term has often been used to refer to physicians’ qualities and approach to practice: their embodiment of trust [28], of competence, compassion, and kindness [12] and “practicing the professional body” [29]. These understandings suggest that embodiment constitutes the manifestation—or attempted manifestation—of physician characteristics vis-à-vis the patient. Embodiment in this sense is wholly contained within and integrated into the being of the physician, a formulation that essentially assumes but fails to specifically address the intersubjective field that exists between the physician and the patient. The current emphasis on understanding the social determinants of health and the contexts of patients’ lives as they relate to their ability to adhere to medical recommendations does little to move away from the view of the Other—the patient—as a being to be acted upon.

In contrast, embodiment may be understood as an intersubjective phenomenon; it is a precondition for intersubjectivity [30]. In writing about body image, Weiss [31] observed, “To describe embodiment as intercorporeality is to emphasize that the experience of being embodied is never a private affair, but is always mediated by our continual interactions with other human and nonhuman bodies.” Csordas [32] in describing intersubjectivity as intercorporeality, noted that “because bodies are already situated in relation to one another, intersubjectivity becomes primary.” Leder [7] views embodiment as inherently relational, involving relationships between persons and between persons and their environments. Jaye [33] suggested that embodiment is “concerned with the lived experience of one’s own body …”, noting that (i)mplicit within the concept of embodiment is a sense of dynamism or constantly shifting meanings and understandings. Embodiment is experienced within particular historical, cultural, political and societal frames, and these experiences are also shaped by gender and race.

In discussing embodiment in the context of education, Latta and Buck also conceive of embodiment as relational in nature.

(E)mbodied teaching and learning is about building relationships between self, others, and subject matter; living in-between these entities … neither subject nor otherness are bound entities; they intermingle. Such intertwining makes it necessary to develop a place for the body in teaching and learning that acknowledges the relational intermingling and flux … a continuous process of reciprocal interaction and modification [34].

“Knowing in any humanly meaningful sense is emergent from and grounded in bodily experience and continuous with the cultural production of meaning” [35]. Research findings lend support to the importance of embodiment. Rizolatti and Craighero [36] have found that because our neural systems recreate what others do and feel, people model others’ behavior or mental states as intentional experiences, a phenomenon that has been referred to as “embodied simulation” [37].

Recent nursing literature emphasizes the importance of efforts to understand the Other, an attuned focus that has been termed “engrossment” [38]. There is a difference between curing the body and healing the embodied person, which includes restoring a patient’s sense of connectedness, control, and wholeness [39].

The question, therefore, becomes whether can one develop a humanistic practice—compassion, empathy—in the absence of embodiment [40,41] and how an understanding of embodiment may be transmitted in the educational context. Knowledge itself does not lead to caring; empathy suggests connection, connection to be forged through embodiment. Embodied care is a process, “an approach to personal and social morality that shifts ethical considerations to context, relationships, and affective knowledge in a manner that can be fully understood only if care’s embodied dimension is recognized. Care is committed to the flourishing and growth of individuals yet acknowledges our interconnectedness and interdependence” [42]. Such care requires movement away from seeing the patient as a body or parts of a body in need of repair to the recognition, acceptance and interaction with the patient as a source of identity and consciousness [43] and the body as a source of meaning [44].

Wisnewski [45] admonished physicians to develop what he termed “sympathetic perception of the patient”, a concept similar to what is referred to here as embodiment. He contrasted the patient’s concern with “being-in-the-world” with the physician’s perception of only a “condition” in front of him or her. In order to convey sympathy, one must perceive sympathetically, “To cultivate sympathetic perception … is to cultivate an appropriate responsiveness to those situations of need we encounter” [45]. Such an attitude cannot be feigned; “emotional attitudes toward others are conveyed beneath the level of self-consciousness,” through inflection and body language [45]; see also [32]. Fuchs [46] recognized the importance of bodily resonance, conceived of as an intuitive understanding of others that occurs in ongoing interactions, often on a pre-reflective level; a process of mutual modifications of bodily and emotional states that takes place as a result of bodily presence [47].

The nonverbal expressions that are revealed through bodily presence constitute a form of knowledge that can be utilized by the health care provider; in essence, the provider “(l)istens within the relationship” with the body [47].

This perspective facilitates the provider’s awareness of preconceptions or biases that they may hold, which may be an obstacle to communication and to a full understanding of the patient and their bodily condition, e.g., in situations where the clinician blames the patient for the illness [48]. This knowledge facilitates the development of an ability to feel empathy and sympathy toward the other.

As Merleau-Ponty observed, perception of behavior in other people and the perception of the body itself by a global corporeal schema are two aspects of a single organization that realize the identification of the self with others. Sympathy would emerge from this. Sympathy does not presuppose a genuine distinction between self-consciousness and consciousness of the other, but rather the absence of distinction between the self and the other. It is the simple fact that I live in the facial expressions of the other, as I feel him living in mine [49].

A recent phenomenological study involving 15 physicians lends support for the importance of embodied communication [50]. Physicians reporting on their use of touch during their clinical experiences with patients have indicated that they relied on patients’ facial expressions and body language to assess the extent to which touch was welcome and used touch as an embodied form of communication to share emotions and to demonstrate presence and empathy.

## 3. Developing Embodied Humanistic Practice

Medical students are taught to navigate their way through a professional culture with its own attitudes, behaviors, rituals, specialized knowledge, and institutional hierarchies [51]. As noted above, there exists a core tension between competency and caring [52], such that technical competence appears to often displace caring. Although many physicians do their best to demonstrate compassion and provide support, this often is insufficient. Sometimes doctors must look through the eyes of those for whom they care in order to better serve their needs. By being “on the other side of the stethoscope” and “wearing a gown”, providers can learn to better empathize with patients and, ultimately, more effectively ease the pain of living with a disease [53].

Such experiences may lead to better communication, more understanding for patients’ difficulties balancing obligations, increased attunement to the emotional aspects of illness, and an understanding of the impact of illness on identity [53]. The question, then, is how this can be achieved without medical students and physicians being patients.

The strategies that are frequently utilized to guide medical students in their development of better communication skills with patients and families and the humanistic practice of medicine are often cognitive in nature, or can be compartmentalized by the learner to be only cognitive, e.g., role playing, journaling. These approaches stand in sharp contrast to the training of osteopaths and homeopaths, which require that they practice skills on each other, requiring that each student feel themselves and the other in their roles as both the provider and the patient.

While we would recognize as ridiculous the suggestion that medical students could be expected to “try out” medication or surgery in themselves, the osteopathy and homeopathy students are expected to experience “being a patient” not only through continued treatment by a qualified practitioner but in the fabric of the organization of training that requires that the students explore their concepts of health and healing and practice their developing skills with each other. During this time, day-to-day absence of the lived body is made visible for the students as they experience new embodied sensations and develop new embodied knowledge. “Playing” the roles of osteopath/homeopath and patient is fundamental to the development of a sense of intersubjectivity and an ability to be reflexive. These new modes of embodied intersubjectivity mean that CAM practitioners are literally and explicitly performing “embodied work”. Carrying out “work on yourself” is commonplace language in CAM communities and is required of both patients and practitioners for a successful therapeutic encounter. [54].

Accordingly, mechanisms to develop caring and empathy must resist superficial patient transactions [43] and focus instead on “somatic cultivation,” i.e., close attention to body [55]. Similarly, Aoki and Ikemi [56] have suggested that focusing, i.e., an “embodied practice where one attends to a bodily felt sense and uses it in understanding oneself and situations” consists of being aware of a sensation, accepting and acting from the sensation, and finding a comfortable distance from that sensation. This requires that the individual assess whether what they are doing is congruent with what they are feeling. Increases in such focusing have been found to be associated with higher levels of empathy [57].

## 4. Arts-Based Approaches

It has been suggested that the use of arts-based methods may be critical to the development embodiment because they encourage individuals to challenge their personal assumptions and dominant cultural narratives [58] develop increased insight, and acknowledge emotions [59]. The extent to which specific modalities are effective in inculcating an understanding of embodiment leading to humanistic practice is unclear due to limited details contained in published reports with respect to theoretical framework; program structure, content, and implementation; participants; short- and long-term outcomes; and behavioral assessments [60].

It has been suggested that forms of performance, in particular, may engage the participant and the viewer viscerally, intellectually, physically, and emotionally. As an example of the impact of performance-focused arts-based approaches, Berland described the impact of filming three individuals with wheelchairs equipped with cameras in the production of the movie, Rolling.

“The three participants in this project taught me to see the world in ways I had never imagined. I do not look at a sidewalk or the incline of a hill as I did before. Steps, doors, building entrances, rooms appear different. I now assess manual and power wheelchairs with a critical eye. The impact, however, is broader than that. I listen more carefully. I consider the time and effort it takes for patients to reach my clinic and how long they have waited before coming to see me with a problem” [61].

Tsaplina [62] has recently advocated the use of puppetry pedagogy as an approach to fostering embodiment. She explained, “The art of animating a puppet trains a deep embodied listening that gives voice and form to material and immaterial presences through the imagination” [62]. She recounts her observations of a student who appears unaware of the concept of or need for embodiment:

“I am observing a pre-med student contend with animating a pair of puppet legs that I built and that I purposefully hinged to bend at the knee in opposing directions. He is agitated by these legs. They will not bend to his will, nor how he imagines legs should be. He wants to be a doctor, maybe a psychiatrist. He does not yet understand that his struggle to force these legs to be what they are not is possibly one of the most important things he can learn about the practice of medicine, care and healing” [62].

Without a sense of embodiment, an awareness of breath, of body, of imagination and intersubjectivity, the student is unable to see the puppet as a whole and as it is, but is only able to imagine what he thinks it should be and should do. The puppet challenges the student to be with, rather than do to.

As Tsaplina notes, puppetry practice holds a unique capacity to illuminate how bodies and worlds are interfaces that become each other, continually. This becoming allows for knowledges and articulations to arise by growing our sensitivity and ability to experience and discern diverse phenomena. What is critical to highlight is that the erasure or elimination of this mutual becoming in the name of “unaffected objectivity” is possible only by the application of power by one body over another, thereby rendering bodies silent through force. [62].

Drama also offers the possibility of developing this sense of relational vulnerability or attunement. Acting in a role provides an opportunity for learners to understand the affective components of an interaction, become more self-aware, and develop empathy [63]. A review of literature relating to the effectiveness of arts-based interventions in medical education concluded that of all of the modalities reviewed—mixed arts, poetry, film, prose, visual arts, and performing arts—only performing arts evidence some effect on the development of positive attitudes that include an increase in empathic feelings [60].

Drama must be distinguished from role play and simulations, which are often used in medical education as an opportunity to practice specific skills. In contrast, “the art of drama is used to illuminate some truth about the world” [64]. Drama includes content, theme, substance, subject matter, and curriculum, offering participants an opportunity to learn about themselves and explore situations from multiple perspectives, their own vulnerabilities, and the complexities of human behavior. Theater can present ambiguities, complexities, and contradictions, challenging the students to identify not only such issues, but their potential resolutions as well [65]. Theater is fundamentally a rehearsal for life [66].

The MEET (Medical Education Empowered by Theater) serves as one example of a drama intervention designed to nurture the cognitive, emotional, and volitional components of empathy [65]. The improvisational exercises that comprise the program involve both body and emotions, recognizing that they are critical elements of empathy. Exercises are followed by a debriefing. One scenario that is used involves a pregnant teenage girl who is waiting in the examination room for a prenatal examination with her mother. While she and her mother are arguing, the supervisor enters with five medical students. The supervisor is dismissive of the girl. The five students display different reactions to the supervisor’s instructions, with some proceeding to conduct the examination, some remaining silent. Students’ narrative evaluations of the program indicated that the course reminded them of the importance of remaining self-aware and to focus on what is happening, rather than accepting situations and events as ordinary and normal.

Scholars have recently noted the underrepresentation in medical education of dance as an approach to the development of embodied awareness [67]. It has been suggested that dance may lead to heightened self-awareness and physical presence and may provide an additional way of knowing.

## 5. Virtual Reality Training

Li and colleagues have proposed using a virtual reality game application to foster students’ close attention to the body [68], noting that this strategy has been used in efforts to foster empathy among medical students [69,70,71,72] and facilitate embodiment [73]. They proposed introducing learners to realistic scenarios involving Parkinson’s disease and requiring that the learners complete specified tasks under the effects of a simulated tremor. The tasks include using a telephone, picking up and putting down pills in the correct order, turning off an alarm clock, showering, brushing teeth, and preparing a sandwich [68]. At the time of this writing, the application has been tested and was ready for classroom use, but its effectiveness in achieving the desired aims is not yet available.

## 6. Implications of Embodied Practice

This article has focused on the rationale for the development of a consciously embodied approach among medical students and the need for educational interventions to encourage this development. Teaching strategies designed to bring awareness to and encourage embodied attunement provide opportunities for learners to challenge their personal assumptions and the dominant cultural narratives, and to reframe their perspective such that they are able to encounter the whole person, rather than viewing the patient as a disembodied entity, body part, or condition. As such, it challenges providers’ understandings of normality and of viewing differently abled/disabled persons as “others” or as not normal or not healthy [74]. The heightened awareness that comes from an embodied approach is intended to facilitate the development of empathy and humanistic practice. Embodiment as a foundation for humanistic practice necessarily entails authenticity—it is not something that can be manufactured and checked off on a list of qualities to be displayed as a means of encouraging patient adherence or disclosure and giving the illusion of shifting power.

The discussion focused on several strategies commonly utilized in medical education to foster empathy and humanistic practice, e.g., arts-based approaches and virtual reality training. An understanding of the extent to which a specific approach effectuates behavioral change will require additional research and clear descriptions of the underlying theoretical approach; program structure, content, and delivery; and short- and long-term attitudinal and behavioral changes.

## Data Availability

Not applicable.
